# Targeting Histone Deacetylases in Myeloid Cells Inhibits Their Maturation and Inflammatory Function With Limited Effects on Atherosclerosis

**DOI:** 10.3389/fphar.2019.01242

**Published:** 2019-10-29

**Authors:** Rosario Luque-Martin, Jan Van den Bossche, Rebecca C. Furze, Annette E. Neele, Saskia van der Velden, Marion J.J. Gijbels, Cindy P.P.A. van Roomen, Sharon G. Bernard, Wouter J. de Jonge, Inmaculada Rioja, Rab K. Prinjha, Huw D. Lewis, Palwinder K. Mander, Menno P.J. de Winther

**Affiliations:** ^1^Department of Medical Biochemistry, Experimental Vascular Biology, Amsterdam Cardiovascular Sciences, Amsterdam UMC, University of Amsterdam, Amsterdam, Netherlands; ^2^Amsterdam UMC, Vrije Universiteit Amsterdam, Department of Molecular Cell Biology and Immunology, Amsterdam Cardiovascular Sciences, Cancer Center Amsterdam, Amsterdam, Netherlands; ^3^Immuno-Epigenetics, Adaptive Immunity Research Unit, GSK Medicines Research Centre, Stevenage, United Kingdom; ^4^Department of Pathology and Department of Molecular Genetics, CARIM, University Maastricht, Maastricht, Netherlands; ^5^Tygat Institute for Liver and Intestinal Research, Amsterdam UMC, University of Amsterdam, Amsterdam, Netherlands; ^6^Institute for Cardiovascular Prevention (IPEK), Ludwig Maximilians University, Munich, Germany

**Keywords:** histone deacetylase, atherosclerosis, therapeutic targeting, monocyte, macrophage maturation

## Abstract

Monocytes and macrophages are key drivers in the pathogenesis of inflammatory diseases. Epigenetic targets have been shown to control the transcriptional profile and phenotype of these cells. Since histone deacetylase protein inhibitors demonstrate profound anti-inflammatory activity, we wanted to test whether HDAC inhibition within monocytes and macrophages could be applied to suppress inflammation *in vivo*. ESM technology conjugates an esterase-sensitive motif (ESM) onto small molecules to allow targeting of cells that express carboxylesterase 1 (CES1), such as mononuclear myeloid cells. This study utilized an ESM-HDAC inhibitor to target monocytes and macrophages in mice in both an acute response model and an atherosclerosis model. We demonstrate that the molecule blocks the maturation of peritoneal macrophages and inhibits pro-inflammatory cytokine production in both models but to a lesser extent in the atherosclerosis model. Despite regulating the inflammatory response, ESM-HDAC528 did not significantly affect plaque size or phenotype, although histological classification of the plaques demonstrated a significant shift to a less severe phenotype. We hereby show that HDAC inhibition in myeloid cells impairs the maturation and activation of peritoneal macrophages but shows limited efficacy in a model of atherosclerosis.

## Introduction

Emerging evidence suggests that epigenetics plays a crucial role in regulating immune cell function and may therefore offer many potential therapeutic opportunities for immune-mediated inflammatory diseases. In recent years, the identification of selective inhibitors of epigenetic enzymes and reader proteins has advanced our understanding of chromatin regulation of gene expression leading to renewed therapeutic efforts to reduce disease progression (Tough et al., 2016; Tough and Prinjha, 2017).

Histone deacetylases (HDAC) are a family of proteins that remove acetyl groups from lysine residues on histone tails and other proteins. The removal of these acetyl groups from histones causes DNA to be more compact, leading to a decrease in gene expression. There are 18 HDAC subtypes within the HDAC family that are subdivided into four classes (I, II, III, and IV) based on their homology to yeast proteins (Koeneke et al., 2015). HDACs in monocytes and macrophages are involved in multiple processes, from maturation to inflammatory response (Das Gupta et al., 2016). The classical inhibitors for these proteins broadly target classes I, II, and IV, which include 11 HDACs (New et al., 2012).

Currently, the use of inhibitors in the clinic is limited to oncology patients due to side effects (Rius and Lyko, 2011; McClure et al., 2018; Suraweera et al., 2018; Banik et al., 2019). Since the inhibition of HDACs offers great potential in several immune-mediated inflammatory diseases (Schotterl et al., 2015; Angiolilli et al., 2017; Cao et al., 2019), the specific targeting of immune cells with inhibitors of epigenetic enzymes may be key to success in non-oncology patients.

Carboxylesterase (CES) enzymes transform membrane-permeable esters into charged acids that are less able to cross the membrane (Imai, 2006). CES1 expression in humans is restricted to hepatocytes and cells of the mononuclear myeloid lineage, such as monocytes and macrophages (Su et al., 2004; Li et al., 2005). Based on this expression pattern, small molecules with an esterase-sensitive motif (ESM) are selectively hydrolyzed by CES1, enabling specific targeting of these cells. The ester-drug leads to the generation and retention of the charged acid, which is also pharmacologically active, within CES1-expressing cells. For instance, the combination of ESM technology with an HDAC inhibitor results in an increase of acetylation levels specifically in monocytes (Needham et al., 2011).

The inhibition of HDAC enzymes has shown wide-ranging anti-inflammatory effects (Falkenberg and Johnstone, 2014; McClure et al., 2018) with demonstrated efficacy in mouse models of inflammatory diseases (Lin et al., 2007; Cao et al., 2014; Hoeksema et al., 2014; Van den Bossche et al., 2014). Furthermore, monocytes and macrophages have an important role in the development and initiation of atherosclerosis (Moore and Tabas, 2011; Swirski et al., 2016; Tabas and Bornfeldt, 2016). Atherosclerosis is a lipid-driven disease that involves chronic inflammation. Monocytes and macrophages detect and phagocytose oxidized low density lipoproteins (oxLDL), becoming foam cells and acquiring a pro-inflammatory phenotype (Moore et al., 2013; Chistiakov et al., 2017). Modulating this phenotype should have beneficial effects in the outcome of the disease.

Based on the importance of myeloid cells in atherosclerosis and the efficacy seen with HDAC inhibitors in models of inflammatory diseases, we wished to evaluate whether HDAC inhibition in myeloid cells would be sufficient to drive efficacy. In our studies, we used a previously characterized molecule, CHR-4487 (ESM-HDAC528) (Needham et al., 2011). A related HDAC inhibitor also using ESM technology (Tefinostat) is being evaluated for efficacy in myeloid oncology indications (Zabkiewicz et al., 2016; Knapper et al., 2018). However, the application of ESM technology outside of oncology therapies has not been fully explored. In our studies, we tested whether ESM-HDAC528 targeting would deliver efficacy in a model of atherosclerosis. We found that compound modulated the pro-inflammatory phenotype and maturation of macrophages, with limited effect on reducing severity of plaques in atherosclerosis but no significant improvement in other disease parameters.

## Materials and Methods

### Compounds

The compound used in the studies was ESM-HDAC528 (also termed CHR-4487) described in the work of Needham et al., 2011. The structure of the compound is shown in **Supplementary Figure 1A**. For *in vitro* work, the compound was dissolved in DMSO and used at a range of concentrations: 10, 50, and 100 nM, and also 1,000 and 10,000 nM for viability studies. For the *in vivo* studies, the compound was used at 3 mg/kg. The compound was dissolved in PBS without calcium and magnesium (PBS -/-), 5% DMSO and 11.25% cyclodextrin. 100 μl of either vehicle control or compound were injected intraperitoneally (i.p.) daily for 4 days for the thioglycollate model and 4 for weeks for the atherosclerosis model.

### Animals

The human CES1 transgenic mouse (*CES1/Es1e^lo^*) was generated by Genoway (Lyon, France) from C57BL/6 mice by targeted insertion of the expression cassette into the expression permissive *hprt* locus on the X chromosome by homologous recombination. Expression of the *CES1* transgene was driven by the human CD68 promoter, which has previously been shown to direct transgene expression in macrophages of transgenic mice (Gough et al., 2001). These mice were then cross-bred with a naturally plasma esterase-low *Es1e^lo^* mouse (obtained from Jackson Labs USA: strain 000785 - B6;D2-a *Ces1c*^e^/EiJ) at Charles River (Margate, UK). From here on, these animals will be referred to as “transgenic mice” or “*CES1/Es1e^lo^*”. Control C57BL/6 wild-type (WT) mice were used in the *in vitro* experiments. In the acute study, twelve 10-week male *CES1/Es1e^lo^* mice were divided in filter-top cages and injected with thioglycolate. Mice were divided in two groups (n = 6 per group) and injected either with 3 mg/kg ESM-HDAC528 or vehicle *via* intraperitoneal (i.p.) injection daily from the day of the thioglycolate injection. On day 3, blood was collected 3 h after i.p. injection, and on day 4, mice were sacrificed 24 h after the last injection for collection of blood and peritoneal cells (PECs).

For atherosclerosis experiments, we made use of low-density lipoprotein receptor knock-out mice (*ldlr*^-^/^-^), which are prone to develop atherosclerosis. *Ldlr*^-^/^-^ mice (C57BL/6 non *Es1e^lo^*) were obtained from Jackson laboratories. A bone marrow transplantation (BMT) was performed by transferring bone marrow from *CES1/Es1e^lo^* mice provided by GlaxoSmithKline into the *ldlr^-/-^* mice. Forty 10-week-old female *ldlr*^-^/^-^ mice were allocated to filter-top cages and provided with water containing neomycin (100 mg/L, Sigma, Zwijndrecht, Netherlands) and polymyxin B sulphate (60,000 U/L, Invitrogen, Bleiswijk, Netherlands) from 1-week pre-BMT until 5 weeks post-BMT. The animals received 2x6 Gy total body irradiation on two consecutive days. Bone marrow from *CES1/Es1e^lo^* mice was resuspended in RPMI-1640 (Gibco, Breda, Netherlands) with 5 U/ml heparin and 2% heat inactivated FCS (Gibco, Breda, Netherlands) and 10^7^ cells were injected intravenously per irradiated mouse. BMT efficiency was determined by qPCR for relative presence of the LDL receptor on DNA isolated from blood (GE Healthcare, Eindhoven, Netherlands). One mouse was excluded from the analysis due to inefficient BMT (<85%). Five weeks after the BMT, the mice were put on a high-fat diet (HFD) (0.15% cholesterol, 16% fat, Arie Blok Diets, Netherlands) for 10 weeks. In week 5, mice were divided in two equal groups by randomization based on weight, cholesterol, and triglyceride levels. One group received 3 mg/kg ESM-HDAC528 and the other received vehicle daily via i.p. dosing for 4 weeks. On week 9, 7 days prior to sacrifice, blood was taken 3 h after i.p. injection of ESM-HDAC528 and on week 10, on the day of the sacrifice, 24 h after i.p. injection of the compound to perform flow cytometry analysis on the blood. After sacrifice, each animal’s heart was excised and frozen in Tissue-Tek (DAKO, Eindhoven, Netherlands) for histology. Two mice were sacrificed before the end of experiment as they reached humane endpoints. One additional mouse was excluded from the analysis due to insufficient tissue quality. A total of 17 mice from ESM-HDAC528 group were compared to 19 mice from the vehicle group for the histological analyses and 18 versus 19 for the flow cytometry experiments, where mice with low number of total events were also excluded.

All animal experiments were conducted at the University of Amsterdam and approved by the Committee for Animal Welfare of the Academic Medical Center, University of Amsterdam (permits: DBC242 and 103169-2). All animal studies were ethically reviewed and carried out in accordance with European Directive 2010/63/EEC and the GSK Policy on the Care, Welfare and Treatment of Animals.

### Bone Marrow-Derived Macrophage Culture and Functional Study

Bone marrow was isolated from femurs and tibia of *CES1/Es1e^lo^* and WT mice by flushing with RPMI-1640. The cells were cultured in RPMI-1640 with 25 mM HEPES and 2 mM L-glutamine, which was supplemented with 10% FCS, penicillin (100 U/ml), streptomycin (100 mg/ml), and 15% L929-conditioned medium as a source of M-CSF for 8 days. On day 8, cells were stimulated with LPS alone (10 ng/ml) or LPS (10 ng/ml) plus IFN-γ (100 U/ml) or left unstimulated for 24 h. Supernatants were collected and IL-6, IL-12(p40), and TNF were quantified by ELISA in accordance with the supplier’s protocols (Life Technologies). Nitric oxide (NO) production was measured by NO_2_ quantification by the Griess reaction. To measure viability, the BMDMs from transgenic mice were pretreated for 30 min with ESM-HDAC528 at 10, 100, 1,000, or 10,000 nM. Afterwards BMDMs were left untreated or stimulated overnight with 20 μg/ml 7-ketocholesterol (7KC; Sigma), 50 μg/ml ox-LDL or 10 μg/ml 25-hydroxycholesterol (25OHC; Sigma) and stained with propidium iodide (PI)/Annexin V-Alexa-Fluor647 according the manufacturer’s instructions (Invitrogen). The percentage of viable macrophages (Annexin V-/PI-) was measured using a FACS Canto II.

After overnight ESM-HDAC528 pretreatment at 10 or 100 nM and DiI-oxLDL (Biotrend) treatment (3 h, 10 μg/ml), Dil-oxLDL uptake was measured by flow cytometry. Oxidized LDL uptake by BMDMs from transgenic mice was measured by flow cytometry. For lipid staining, BMDMs were pretreated with the inhibitors for 30 min, stimulated with 50 μg/ml oxLDL (BTI) for 24 h, and stained with LipidTOX Red (Invitrogen) according to the manufacturers’ instructions. The median fluorescence intensities (MFI) were calculated with FlowJo software version 10.4.2.

### Peritoneal Macrophages

Four days prior to the sacrifice, mice were injected intraperitoneally with 1 ml thioglycolate medium (3%, Fisher, Bleiswijk, Netherlands). Upon sacrifice, the peritoneum was flushed with 10 ml ice cold PBS and PECs were collected as described previously (Neele et al., 2017). Flushed thioglycolate-elicited cells were cultured at a density of 100,000 cells/well in 100 µl in 96-well tissue culture plates (Greiner Bio-One, alphen a/d Rijn, Netherlands) in RPMI-1640 containing 25mM HEPES, 2mM L-glutamine, 100 U/ml penicillin, and 10% FCS (all Gibco, Breda, Netherlands). After 3 h adherence, non-adherant cells were washed away and the adherent cells were left either unstimulated or stimulated for 24 h with LPS (10 ng/ml) alone, LPS (10 ng/ml) plus IFN-γ (10 U/ml), or 24 h with IL-4 (20 ng/ml). Supernatants were collected and IL-6, IL-12(p40)/IL-12(p70), and TNF were quantified by ELISA in accordance with the supplier’s protocols (Life Technologies). NO production was measured by NO_2_ quantification in a Griess reaction. Cells were harvested using 1x Citrate from a 10X stock solution (1.35M potassium chloride, 0.15M sodium citrate, dilute in 100 ml milliQ and autoclaved) for 5 min at 37°C; the reaction was stopped by adding PBS-/- and cells were detached and washed twice with FACS buffer. Fc receptors were blocked with CD16/CD32 blocking antibody (1:100, eBioscence) in FACS buffer and cells were stained with appropriate antibodies (**Supplementary Table 2**) for 30 min at RT. Cells were then washed with FACS buffer and fluorescence was measured with a CytoFLEX flow cytometer and analysed with FlowJo software version 10.4.2. Cells were gated by excluding doublets, then selecting the macrophages based on FSC-A/SSC-A parameters. Positive peaks for markers were defined based on isotype control antibodies and the MFI was determined. This method was also used to measure the expression of alternative activation markers (PDL2, CD71, CD206, CD301) *in vitro* in BMDMs from transgenic mice following treatment with ESM-HDAC528 at concentrations of 10, 50, and 100 nM and with IL-4 (20 ng/ml) for 24 h.

PECs were used immediately post-isolation to quantify mature peritoneal macrophages (PEMs) and intracellular lysine acetylation levels within those cells. Lysine acetylation was determined using the same protocol as for blood, minus the red blood lysis step. To evaluate maturation markers, cells were washed with FACS buffer and then stained with appropriate antibodies (**Supplementary Table 3**) for 30 min at RT and Fc receptors were blocked with CD16/CD32 blocking antibody (1:100, eBioscence) in FACS buffer. Cells were then washed with FACS buffer and fluorescence was measured with a CytoFLEX flow cytometer and analysed with FlowJo software version 10.4.2. After removing the doublets, macrophages were defined as CD11b^+^ and F4/80^+^ and then maturation markers Ly6C and CD64 were measured in these populations.

### Intracellular Acetylation Flow Cytometry and Triglyceride/Cholesterol Measurement

100 μl of blood were withdrawn from mice at 3 h and 24 h after i.p. injection of ESM-HDAC528. The blood was collected in tubes containing sodium heparin. For the 3 h time point, mice were injected with 3 mg/kg ESM-HDAC528 and their food was restricted for 3 h in order to get an accurate measurement of triglycerides and cholesterol. 50 μl of blood were centrifuged (10 min, 4°C, 2,000 rpm) to separate the plasma from blood cells. Plasma cholesterol and triglyceride levels were enzymatically measured according to the manufacturer’s protocol (Roche, Woerden, Netherlands). 50 μl of blood was used for flow cytometry to measure intracellular acetylation at 3 h and 24 h. The blood was mixed 1:1 with PBS -/- and stained with cell surface marker antibodies for 30 min on ice (**Supplemental Table 1**). Red blood cells were lysed and cells were fixed by using BD FACS Lyse/Fix solution following the manufacturer’s instructions (BD Pharmingen). After washing the cells twice with FACS buffer (0.5% BSA, 0.01% NaN_3_ in PBS), cells were permeabilized using Human FoxP3 buffer following manufacturer’s instructions (BD Pharmingen) and stained with an antibody for acetylated lysine (PanAck, Biolegend) for 30 min at RT. Cells were washed twice and resuspended in FACS buffer. Data were acquired using a BD Canto II and analysed with FlowJo software version 10.4.2. The cells were gated by excluding doublets, and then Ly6G^+^ neutrophils were distinguished from monocytes, B, and T cells. Monocytes (CD11b^+^/CD115^+^) were distinguished from lymphocytes. Lymphocytes were further separated in B cells (B220^+^/CD3^-^) and T cells (B220^-^/CD3^+^). The MFI was determined from the positively stained cells (following FMO and isotype control corrections).

### Histochemistry

Atherosclerotic lesions from the heart were cut into 7 mm sections on a Leica 3050 cryostat at −25°C. Cross area sections of 42 mm were stained with toluidine blue (0.2% in PBS, Sigma-Aldrich, Gillingham, UK) to determine lesion size. Total lesion size per section was measured using Adobe Photoshop CS4. Lesion severity was scored (0, 1, 2, 3, 4, 5) by an experienced pathologist as no lesion (score 0) early (intimal xanthoma, scores 1, 2), moderate (pathological intimal thickening, score 3) and advanced (fibrous cap atheroma, scores 4, 5), as described elsewhere (Kanters et al., 2003). Sirius red staining was performed for 30 min to measure collagen content (0.05% direct red in saturated picric acid, Sigma, Zwijndrecht, Netherlands). Images were obtained using a Leica DM3000 microscope and quantified with Adobe Photoshop CS4 where collagen was quantified as the percentage of total lesion size. For immunohistochemistry, slides were fixed in acetone and blocked with Avidin/Biotin Blocking Kit (Vector Laboratories, Burlingame, USA). Hereafter, cells were incubated with MOMA-2 (1:4000, AbD Serotec, Uden, Netherlands) to stain for macrophages, ER-MP58 (1:200, AbD serotec, Uden, Netherlands) for infiltrating monocytes. Necrosis area was measured based on toluidine blue staining by a pathologist and corrected for total plaque size.

### Human Whole Blood Intracellular Acetylation Measurement

All donors provided written informed consent for the use of their samples, and the collection and use of the samples received Institutional Review Board approval. Blood from healthy volunteer donors was collected into tubes containing sodium heparin anticoagulant. 140 μl of blood was treated with compound for 4 h at 37°C after which samples were fixed and lysed for 15 min using FACS lysing solution (BD Pharmingen). Cells were washed with FACS buffer and Fc receptors were blocked using human IgG (Sigma) for 15 min at RT. Samples were stained at RT for 30 min with anti-CD66 (BD Pharmingen 551479) and anti-CD14 (BD Pharmingen 555399), to identify neutrophils and monocytes, after which samples were washed twice in FACS buffer and permeabilized for 30 min at RT using nuclear permeabilization buffer (Biolegend). Samples were then washed once and resuspended in nuclear permeabilization buffer containing anti-acetylated lysine antibody (Biolegend 623404) or a matched isotype control (R&D Systems IC0041P) and incubated at RT for 30 min (**Supplementary Table 4**). Samples were washed twice in PBS and sample data were acquired using the BD FACS Canto II Flow Cytometer with FACS Diva (BD BioSciences software version 6.1.3.). Cells were gated by excluding doublets and neutrophils and monocytes identified. The remainder of nonstained viable cells were defined as lymphocytes. The MFI (median fluorescent intensity) of acetylated lysine within each population was determined.

### Statistical Analysis

Data represent the mean ± standard error of the mean (SEM). Differences between groups were analyzed using an unpaired student’s *t*-test, two-way ANOVA using Bonferroni post hoc test analysis for grouped analysis or Chi-squared test. *P*-values <0.05 were considered statistically significant. Nonlinear curves for concentration-response studies for the data from human whole blood intracellular acetylation experiments were also generated. Data were analyzed using GraphPad Prism version 5.0 (GraphPad software, La Jolla, California).

To assess plaque severity (ranked 0, 1, 2, 3, 4, 5), an average severity score (based on 2–3 sections per animal) was calculated to give a single value for each animal. A nonparametric Mann-Whitney test was applied using Prism version 5.0 (GraphPad software, La Jolla, California) to determine whether the median score differed significantly between the treatments.

## Results

### ESM-HDAC528 Reduces Pro-Inflammatory Cytokine Production

The mouse orthologue of CES1 significantly differs in distribution of expression and substrate specificity (Berry et al., 2009). Therefore, we utilized transgenic mice containing the human *CES1* gene under the control of the *CD68* promoter. Bone marrow-derived macrophages (BMDMs) from transgenic or WT mice were activated with LPS or LPS/IFNγ in the presence of the targeted HDAC inhibitor (ESM-HDAC528). Structurally, the ESM-HDAC528 compound is composed of an HDAC inhibitor conjugated to an ester group. When the ester group is cleaved from the HDAC inhibitor, by the enzyme CES1, in myeloid cells of the transgenic mice, it accumulates within those specific cells (**Supplementary Figure 1A**).

**Figure 1:**
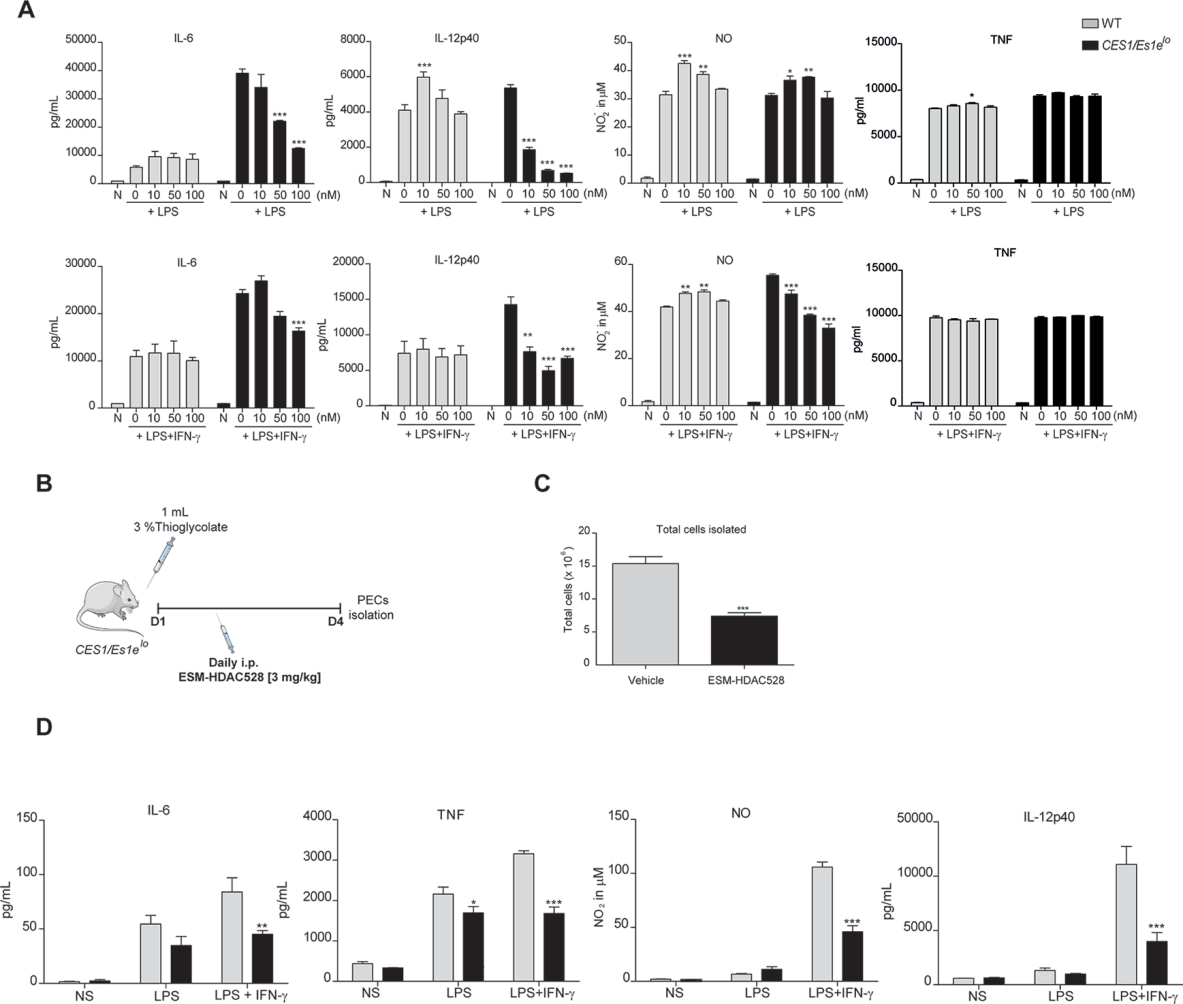
ESM-HDAC528 reduces pro-inflammatory cytokine production both *in vitro* and *in vivo*. **(A)** Cytokine production by BMDMs from *CES1/Es1e*^lo^ mice and WT mice after stimulation with LPS (10 ng/ml) or LPS (10 ng/ml) + IFN-γ (10 U/ml) in the presence of increasing concentrations of ESM-HDAC528 for 24 h. n = 3. **(B)** Design of acute thioglycolate model. Transgenic mice were treated for 4 days with daily i.p. injection of 3 mg/kg ESM-HDAC528 (n = 6) or vehicle (n = 6), on day 4, 24 h after i.p. injection PECs were isolated. **(C)** Total number of cells isolated from the peritoneal lavage in each group n = 6 per group. **(D)** Cytokine production by PEMs isolated from the mice (n = 6) of each group attached and then stimulated for 24 h with LPS (10 ng/ml) or LPS (10 ng/ml) + IFN-γ (10 U/ml). Statistical significance was determined by unpaired *t*-test (C) or two-way ANOVA with Bonferroni correction **(A**, **D)** (p < 0.05). All error bars represent the SEM. ns = *p* value > 0.05, *p value ≤ 0.05, **p value ≤ 0.01, ***p value ≤ 0.001.

After stimulation, we observed a concentration-dependent inhibition of the production of pro-inflammatory mediators (IL-6, IL-12p70, NO) but not for TNF (**Figure 1A**). No inhibition was observed with compound in WT macrophages at these concentrations, likely due to the lack of expression of the human enzyme. Further characterization of functions related to atherosclerosis of BMDMs from the transgenic mice after treatment with ESM-HDAC528 were also performed. Firstly, viability was assessed (**Supplementary Figure 1B**). No effects on viability were observed in the cells after treatment with 10 and 100 nM of ESM-HDAC528. At higher concentrations (1,000 and 10,000 nM), the viability was reduced. Based on these results, subsequent experiments were performed at 10, 50, and 100 nM. The expression of alternative activation surface markers (**Supplementary Figure 1C**) after IL-4 stimulation was determined. No significant changes were observed except for a trend to reduction in the expression of CD206 at higher concentrations. Another important function is lipid uptake (**Supplementary Figure 1D**); in this case, no effects were observed in uptake of oxidized LDL after treatment with ESM-HDAC528.

Prior to *in vivo* studies, intracellular acetylation of white blood cells was determined in human whole blood treated with either the non-targeted, conventional HDAC inhibitor SAHA (suberanilohydroxamic acid) or ESM-HDAC528 (**Supplementary Figures 1E and 1F**). ESM-HDAC528 was more potent than SAHA at increasing intracellular acetylation levels and this phenomenon was selectively observed in monocytes.

To assess if a suppressed macrophage response also manifested *in vivo*, an inflammatory response was initiated in *CES1/Es1e*^lo^ mice by a single i.p. thioglycolate injection (**Figure 1B**). It has previously been demonstrated that ESM-HDAC528 specifically targets circulating monocytes (Needham et al., 2011) and we wanted to extend this observation to PEMs. In this study, mice were injected i.p. daily for 4 days with 3 mg/kg ESM-HDAC528 or vehicle and on day 4 PECs were isolated. The total number of cells isolated from the ESM-HDAC528 group was significantly reduced compared to the vehicle group (**Figure 1C**).

After 3 h attachment (to enrich for PEMs), cells isolated from both groups were stimulated *in vitro* with LPS or LPS/IFNγ. Interestingly, PEMs from the ESM-HDAC528 group produced lower levels of pro-inflammatory mediators after activation compared to equal numbers of plated PEMs from the vehicle group (**Figure 1D**). These data indicate that ESM-HDAC528 reduces macrophage activation both *in vitro* and *in vivo*.

### ESM-HDAC528 Modulates the Maturation of Freshly Isolated PEMS and the Expression of Macrophage Activation Markers on Cultured PEMS.

**Figure 2:**
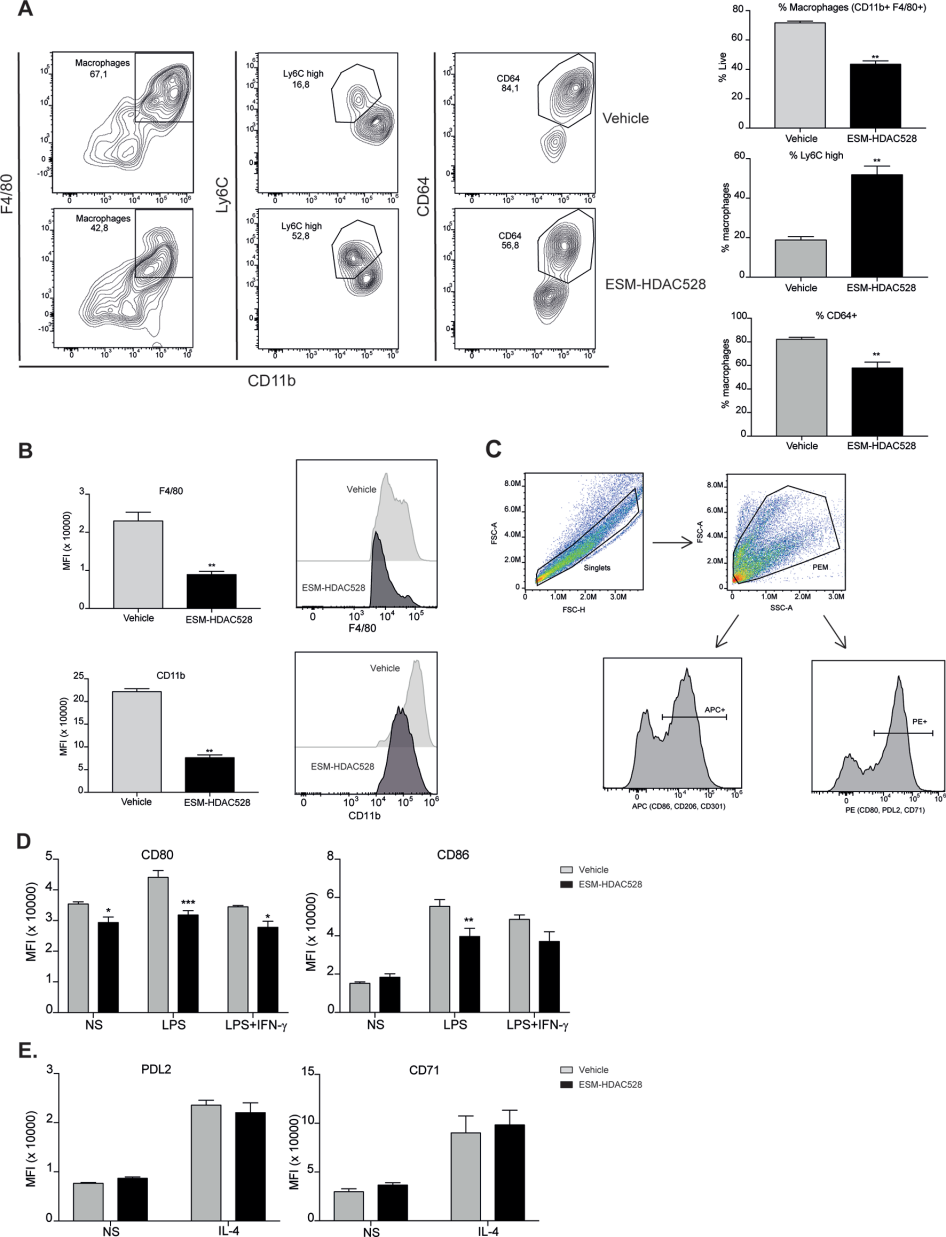
ESM-HDAC528 treatment modulates PEM maturation and surface marker expression. **(A)** Percentage of mature macrophages (CD11b+ and F4/80+), and the maturation markers Ly6C and CD64 within macrophage populations in the freshly isolated cells 24 h after injection n = 6 per group. **(B)** MFI of F4/80 and CD11b in the mature macrophages. n = 6 per group. **(C)** General gating strategy for activation marker expression on PEMs after attachment and 24 h stimulation for activation. Antibodies were conjugated to either APC or PE depending on the panel. **(D)**. MFI of the positive peaks for the pro-inflammatory surface markers in PEMs Isolated, attached and stimulated for 24 h with LPS (10 ng/ml) or LPS (10 ng/ml) + IFN-γ (10 U/ml). n = 6 per group. **(E)** MFI of the positive peaks for the alternative activation surface markers in PEMs attached and stimulated for 24 h with IL-4 (20 ng/ml). n = 6 per group. Statistical significance was determined by unpaired *t*-test **(A** and **B)** or two-way ANOVA with Bonferroni correction **(D** and **E)** (p < 0.05) All error bars represent the SEM. ns = *p* value > 0.05, *p value ≤ 0.05, **p value ≤ 0.01, ***p value ≤ 0.001.

We next wanted to understand whether the effects of an ESM-HDAC inhibitor on cytokine production were due to a change in polarization or maturation. The maturation status was measured in freshly isolated PEMs. Mature PEMs can be defined as a CD11b^+^ and F4/80^+^ population (Misharin et al., 2013; Cassado et al., 2015) (**Figure 2A**). The percentage of this population was different between groups, with a reduction of 28% in the ESM-HDAC528 group compared to vehicle-treated mice. Other markers (Ly6C and CD64) were also measured within the macrophage population (CD11b^+^ F4/80^+^). Ly6C is a monocyte marker expected to be higher in immature macrophages (Robbins et al., 2013), whereas CD64 is expressed in mature macrophages rather than in monocytes (Tamoutounour et al., 2012). The population showed an increased percentage of Ly6C^+^ cells and reduction in CD64^+^ cells which indicates reduced maturation in the PEMs of the ESM-HDAC528 treatment group (**Figure 2A**). Additionally, in cells that were mature (CD11b^+^ and F4/80^+^), the MFI for these maturation markers (CD11b and F4/80) was lower in the ESM-HDAC528 group (**Figure 2B**).

Next, we measured the expression of pro-inflammatory and alternative activation surface markers. The gating strategy used for stimulated cells is shown (**Figure 2C**). After attachment, the cells are expected to be predominantly PEMs. After stimulation, cells were harvested, doublets excluded, and the PEMs were gated based on FSC-A/SSC-A. Surface markers were detected using either PE- or APC-conjugated antibodies. The positive peaks of those markers were defined using an isotype control and the MFI of the markers was determined from the positive population (**Figure 2C**). CD80 expression was significantly decreased on both unstimulated and stimulated PEMs from ESM-HDAC528 treated mice compared to vehicle controls. Furthermore, CD86 expression was significantly lower in LPS-treated PEMs (**Figure 2D**). No effects were observed on PDL2 and CD71, IL-4-induced markers of alternatively activated macrophages (**Figure 2E**). We conclude that ESM-HDAC528 blocks PEM maturation and inhibits the expression of pro-inflammatory markers.

### ESM-HDAC528 Treatment Does Not Affect Lipid Levels in an Atherosclerosis Model

**Figure 3:**
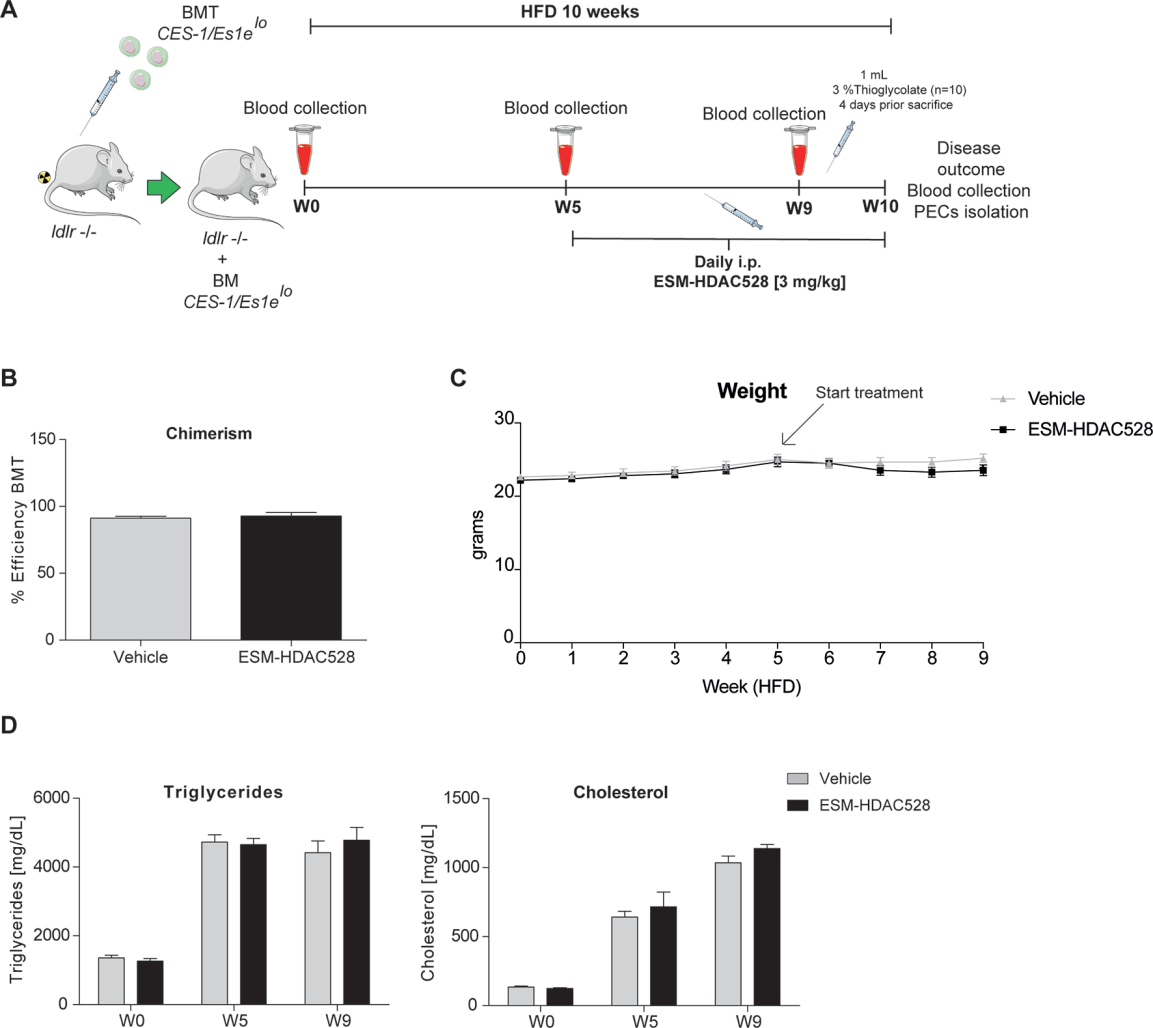
ESM-HDAC528 treatment does not affect clinical features in a model of atherosclerosis. **(A)** Design of the atherosclerosis study. The *ldlr* knockout mice were transplanted with bone marrow from *CES-1/Es1e^lo^* mice. The mice were divided into two groups ESM-HDAC528 (n = 19) and vehicle (n = 19). Mice were on an HFD for 10 weeks. On week 5, the mice were treated with 3 mg/kg ESM-HDAC528 or vehicle by daily i.p. injection. Blood was collected on week 0, 5, 9, and 10. On week 10, mice were sacrificed, the disease outcome was assessed and PECs were isolated. **(B)** Efficiency of the BMT in both groups. n = 19 vehicle vs. n = 18 ESM-HDAC528. **(C)** Weight of the mice from both groups during the study. n = 19 vehicle vs. n = 18 ESM-HDAC528 **(D)** Triglycerides and cholesterol levels of the groups on week 0, 5, and 9. n = 19 vehicle vs. n = 18 ESM-HDAC528. Statistical significance was determined by unpaired *t*-test **(B)** or two-way ANOVA with Bonferroni correction **(C** and **D)** (p < 0.05). All error bars represent the SEM.

The preceding experiments demonstrated that ESM-HDAC528 affects the pro-inflammatory and maturation status of macrophages. We next wanted to test if this would be of benefit in a model of atherosclerosis. In this *in vivo* atherosclerosis study, *ldlr ^-/-^* mice were irradiated and transplanted with bone marrow from *CES1/Es1e^lo^* mice. Mice were fed for 10 weeks on an HFD and treated from week 5 either with 3 mg/kg ESM-HDAC528 or vehicle (**Figure 3A**).

The efficiency of the BMT, measured by chimerism, was equal and above the threshold of 85% for the mice divided between both analysis groups (**Figure 3B**). The mean weight remained similar across the study in both treatment groups (**Figure 3C**). As expected, triglycerides and cholesterol levels increased over the study duration. However, levels of both analyses remained similar in both groups (**Figure 3D**).

### ESM-HDAC528 Increases Acetylation Specifically in Murine Myeloid Cells

**Figure 4:**
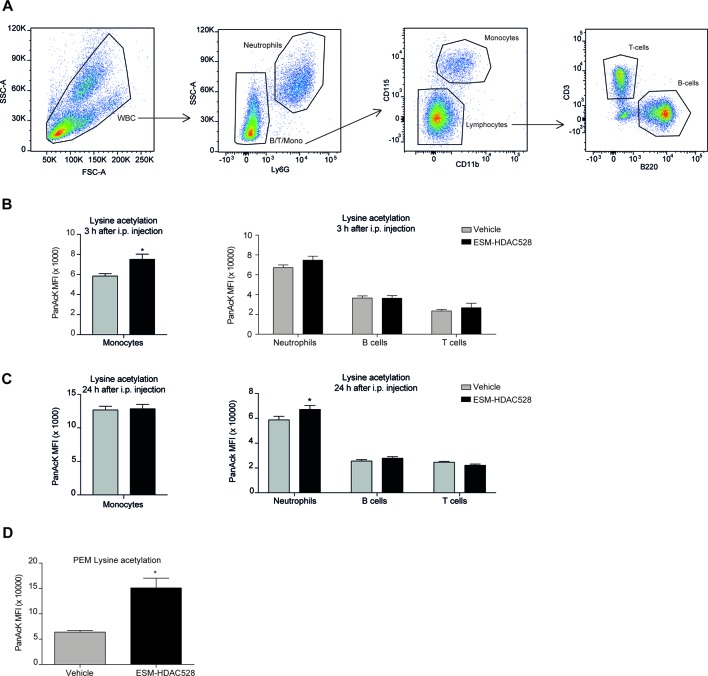
ESM-HDAC528 selectively increases acetylation in myeloid cells. **(A)** Gating strategy for white blood cells after doublet exclusion. **(B)** MFI of intracellular acetylation levels in monocytes and other WBC 3 h after i.p. injection. n = 19 vehicle vs. n = 18 ESM-HDAC528. **(C)** MFI of intracellular acetylation levels in monocytes and other WBC 24 h after i.p. injection. n = 19 vehicle vs. n = 18 ESM-HDAC528. **(D)** MFI for intracellular acetylation levels in fresh PEMs isolated 24 h after i.p. injection. n = 5 per group. Statistical significance was determined by unpaired *t*-test **(B** and **C)** or two-way ANOVA with Bonferroni correction **(B**–**D)** (p < 0.05). All error bars represent the SEM. *p value ≤ 0.05

To confirm the targeted activity of ESM-HDAC528, we measured the levels of acetylation in circulating white blood cells using the gating strategy described (**Figure 4A**). In blood samples collected 3 h after ESM-HDAC528 treatment, there was a significant increase in acetylation in monocytes. The acetylation levels in other immune cells were unchanged compared to the vehicle control, showing the specific targeting of this compound to mononuclear myeloid cells (**Figure 4B**). After 24 h, when compound was no longer systemically detectable, acetylation was modestly increased in neutrophils in this study, although monocyte acetylation had reverted to similar levels in both groups (**Figure 4C**).

We also determined acetylation in freshly isolated PEMs and found higher levels in the compound group compared to the vehicle, demonstrating specificity of the compound not only in monocytes but also in macrophages in this mouse model (**Figure 4D**).

### ESM-HDAC528 Modulates PEM Maturation and Activation to a Lesser Extent in the HFD Atherosclerosis Model

**Figure 5:**
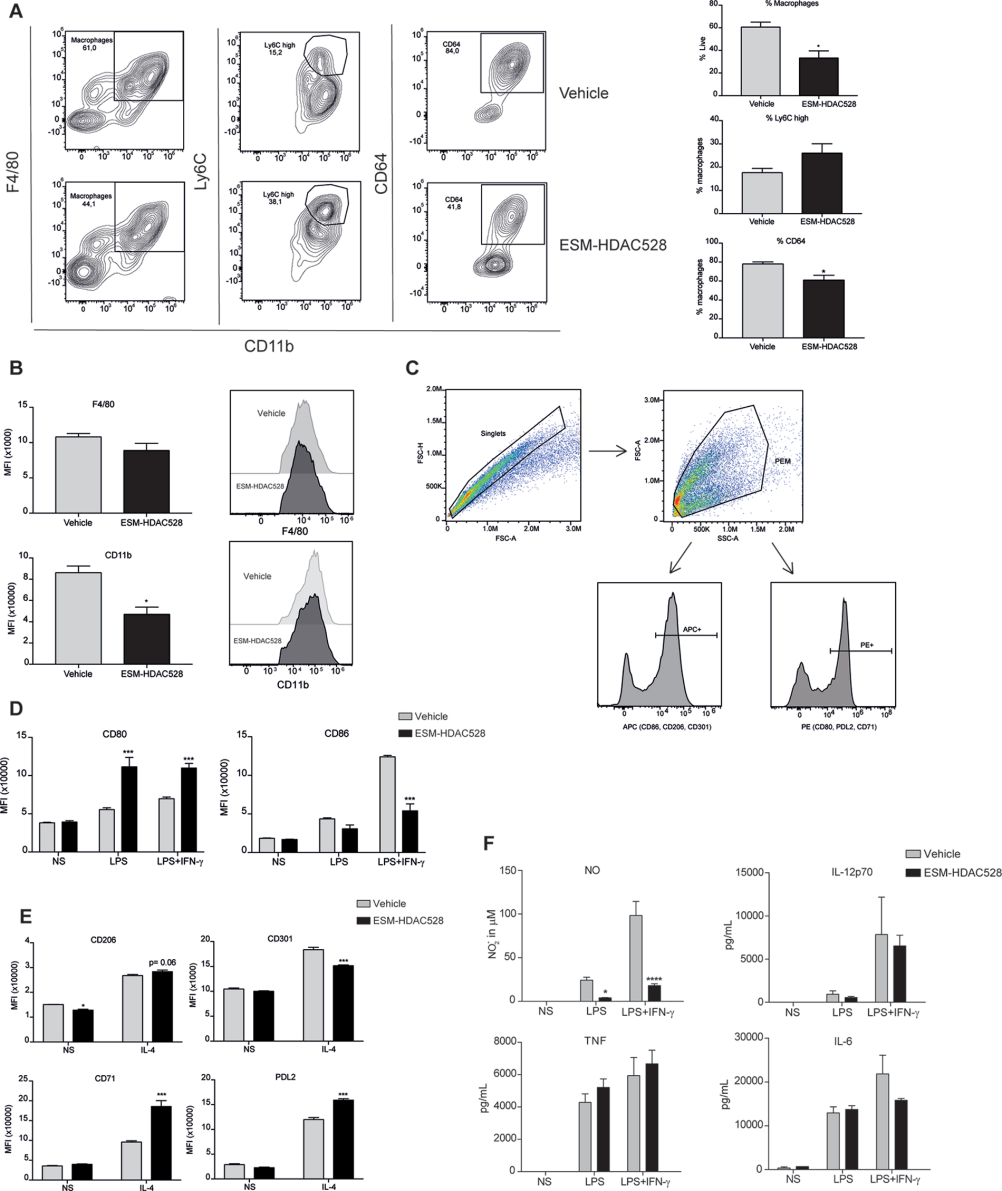
ESM-HDAC528 modulates PEM maturation and activation to a lesser extent in an atherosclerosis model. **(A)** Percentage of mature (CD11b+ and F4/80+) macrophages from freshly isolated PECs 24 h after ESM-HDAC528 injection and of cells expressing Ly6C and CD64 within this population. n = 5 per group. **(B)** MFI of F4/80 and CD11b in the mature macrophages 24 h after injection. n = 5 per group. **(C)** General gating strategy for activation marker expression on PEMs after attachment and 24 h stimulation for activation. Antibodies were conjugated to either APC or PE dependent on the panel. **(D)** MFI of the positive peaks for pro-inflammatory surface markers in PEMs Isolated, attached and stimulated for 24 h with LPS (10 ng/ml) or LPS (10 ng/ml) + IFN-γ (10 U/ml). n = 5 per group. **(E)** MFI of the positive peak for alternative activation surface markers in PEMs attached and stimulated for 24 h with IL-4 (20 ng/ml). n = 5 per group. **(F)** Cytokine production by PEMs isolated from the mice (n = 5) of each group stimulated 24 h with LPS (10ng/ml) or LPS (10ng/ml) + IFN-γ (10 U/ml). Statistical significance was determined by unpaired *t*-test **(A** and **B)** or two-way ANOVA with Bonferroni correction **(D**–**F)** (p < 0.05). All error bars represent the SEM. ns = *p* value > 0.05, *p value ≤ 0.05, **p value ≤ 0.01, ***p value ≤ 0.001.

We wanted to evaluate whether ESM-HDAC528 dampened macrophage maturation and activation in the atherosclerosis model to a similar extent to that seen in the acute model (**Figure 2**). The percentage of mature macrophages (CD11b^+^ F4/80^+^) following thioglycolate administration was lower in the compound-treated group (**Figure 5A**). Within this macrophage population, maturation markers also showed the same trend as previously observed, with CD64 expression being significantly lower in the compound group and a trend for increased Ly6C (**Figure 5A**). The expression levels of the maturation markers CD11b and F4/80 (as assessed by MFI) were significantly lower for CD11b and there was a trend toward a reduction of F4/80 expression within the mature macrophages in the ESM-HDAC528 treatment group (**Figure 5B**). Overall, we observed an effect of the compound on the maturation of macrophages, although the magnitude of change was generally weaker than observed in the acute model.

The gating strategy for the measurement of activation markers was as previously defined (**Figure 5C**). Interestingly, in contrast to our previous observation, in PEMs from atherosclerotic mice stimulated with LPS alone or in addition to IFNγ, an increase of CD80 was seen following ESM-HDAC528 treatment. However, for LPS+IFNγ induced expression of CD86, there was a reduction in the ESM-HDAC528 treated animals (**Figure 5D**). For the alternative activation markers following IL-4 stimulation, there was an increase of CD71 and PDL2 and a reduction of CD301 with ESM-HDAC528 (**Figure 5E**). Pro-inflammatory mediators were also measured, and, except for NO, which was reduced in the ESM-HDAC528 group, there was no significant inhibition of the production of pro-inflammatory mediators by macrophages (**Figure 5F**). In general, ESM-HDAC528 had a reduced ability to inhibit macrophage maturation in this model. Additionally, the effects on pro-inflammatory mediators were milder and polarization markers were inconsistent with inhibiting a pro-inflammatory phenotype.

### Disease Outcome After Treatment With ESM-HDAC528

**Figure 6:**
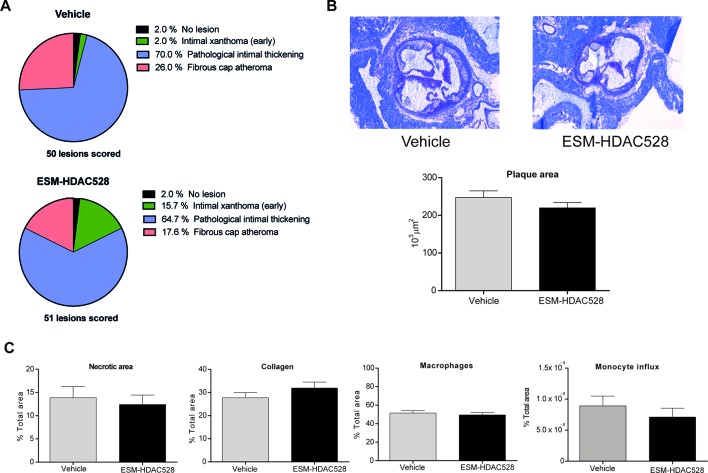
ESM-HDAC528 does not reduce plaque formation. **(A)** Severity of the plaque for the different groups, the plaque is rated according to the morphology. n = 50 lesions in vehicle vs. n = 51 lesions in ESM-HDAC528. **(B)** Plaque size for the different groups. Representative images of the plaque area and measurement of plaque area for the different groups. n = 19 vehicle vs. n = 17 ESM-HDAC528. **(C)** Different disease characteristics: necrotic area, percentage of macrophages, collagen, and monocyte influx in the plaques of the different treatment groups. n = 19 vehicle vs. n = 17 ESM-HDAC528. Statistical significance was determined by unpaired *t*-test **(B** and **C)** or Chi-square test **(A)** (p < 0.05). All error bars represent the SEM.

Considering the characteristics of the macrophages after the treatment with ESM-HDAC528, we wanted to understand the impact on the disease outcome. Therefore, the severity of the atherosclerotic lesions in the mice was scored. We observed a reduction in the percentage of the more severe phenotypes of the plaques (pathological intimal thickening and fibrous cap atheroma) in the ESM-HDAC528 treated mice together with an increase of the less severe phenotype (intimal xanthoma) (**Figure 6A**). The severity scores of all lesions were combined to give a composite score per animal, showing that the animals treated with ESM-HDAC528 had a significantly reduced median severity score upon ESM-HDAC528 treatment compared to vehicle treated mice (ESM-HDAC528 median = 3.00; vehicle median = 3.67; *p* = 0.0163). A trend for reduction in total plaque area was also observed ESM-HDAC528 treated mice (**Figure 6B**).

For the rest of the disease parameters, there were no significant differences between the groups. Nevertheless, the necrotic plaque area and monocyte influx also showed a tendency to be reduced in the atherosclerosis model and the increase in collagen could indicate a more beneficial phenotype (**Figure 6C**). In conclusion, in parallel to effects on maturation of the macrophages, atherosclerotic plaque severity was partially improved by the ESM-HDAC528 treatment in this model.

## Discussion

Macrophages play a role in virtually every stage of atherosclerosis and reshaping their dysregulated activation is considered to be the holy grail of macrophage therapeutic targeting (Sica and Mantovani, 2012). The Canakinumab Anti-inflammatory Thrombosis Outcomes Study (CANTOS) recently delivered clinical data demonstrating that inflammation is a key driver of atherosclerosis (Ridker et al., 2017). Therefore, targeting macrophage-mediated inflammation has emerged as an attractive approach for atherosclerosis therapy. Meanwhile, it has become increasingly clear that epigenetic mechanisms are critical regulators of inflammatory responses. Histone deacetylases that regulate the acetylation status of histones and non-histone proteins are of high interest since broad-spectrum HDAC inhibitors are well documented to decrease inflammation and disease severity in multiple diseases (Neele et al., 2015). Moreover, inhibition of HDACs in macrophages has beneficial athero-protective effects *in vitro* (Van den Bossche et al., 2014) but their progression as a potential atherosclerosis therapy was prevented by the observation that the broad-spectrum HDAC inhibitor Trichostatin A (TSA) unexpectedly increased plaque size in a mouse model of atherosclerosis (Choi et al., 2005). However, this could be due to negative effects of TSA on other cell types that are known to affect atherosclerosis such as endothelial cells and smooth muscle cells (Rössig et al., 2002).

Therefore, we reasoned that inhibiting HDACs specifically in macrophages and monocytes would be beneficial in an atherosclerosis setting. To achieve this, we used an ESM-conjugated HDAC inhibitor which is selectively hydrolyzed into a charged molecule and retained within monocytes and macrophages by human carboxylesterase-1 (CES1) (Needham et al., 2011). Accordingly, ESM-HDAC528 had no inhibitory effect on BMDMs from WT mice but efficiently inhibited inflammatory responses in BMDMs that were derived from transgenic mice that expressed human CES1 driven by the monocyte/macrophage-specific CD68 promoter (*CES1/Es1e^lo^*). ESM-HDAC528 exhibited monocyte-specific activity (lysine acetylation) in human white blood cells. Moreover, we found that ESM-HDAC528 was more potent than the conventional non-targeted HDAC inhibitor SAHA in these cells.

*In vitro* data showed decreased levels of cytokine production in transgenic BMDMs in contrast to WT BMDMs where no decreases were observed. Interestingly, in the case of IL-12p40, NO, and, to a much lesser extent, TNF, a significant induction was observed in WT cells. This phenomenon is not understood and could be addressed in future work with more extended concentration ranges to explore the potential of biphasic responses.

After validating our approach *in vitro*, we next confirmed the efficacy of ESM-HDAC528 *in vivo* and that i.p. injection of the drug into *CES1/Es1e^lo^* mice efficiently reduced the LPS (+/- IFNγ)-induced production of IL-6, TNF, IL-12, and NO. Interestingly, the total number of PECs and macrophages isolated from ESM-HDAC528-injected mice was reduced and these cells appeared less mature as evidenced by increased Ly6C and decreased CD11b, F4/80, and CD64 expression. Since these distinct ESM-HDAC528-mediated effects could potentially dampen atherosclerosis progression, we next assessed the effect of this drug on atherosclerosis in *ldlr^-/-^* mice that were transplanted with *CES1/Es1e^lo^* bone marrow. Acetylation levels in monocytes and PEMs were increased in the ESM-HDAC528-treated group and this was accompanied by reduced macrophage activation. Yet, the effects of HDAC inhibition on inflammatory and maturation endpoints in PEMs were less pronounced in these hypercholesterolemic mice. Although ESM-HDAC528 treatment did not significantly affect plaque size, these plaques were classified as less severe histological phenotypes, with the change being statistically significant and consistent with at least a partial impact on an important disease outcome.

One of the outstanding questions from our observations is how HDAC inhibition impairs monocyte to macrophage differentiation and inflammatory responses, and why the latter effect is less pronounced in a hypercholesterolemic environment. It should be noted that, in this BMT model, ESM-HDAC528 would not be targeted to non-bone marrow derived lineages of macrophages which would not express human CES1. This could explain the limited efficacy seen in the atherosclerosis model. Additionally, it is well described that cell fate decisions within the hematopoietic system are regulated by epigenetic mechanisms and distinct HDACs were shown to be implicated in myeloid development (reviewed in (Das Gupta et al., 2016)). Specific HDACs are differentially regulated and expressed in response to environmental factors, and while HDAC inhibitors mediate anti-inflammatory effects *via* a wide range of mechanisms, they can also amplify inflammatory responses in macrophages. For example, HDAC6 normally acts as a transcriptional activator of the anti-inflammatory cytokine IL-10 and consequently HDAC6 inhibition or genetic knock-down diminishes IL-10 secretion (Cheng et al., 2014). As such, ESM-HDAC528 could potentially inhibit distinct HDACs in normal versus hypercholesterolemic mice and this might explain why broad spectrum HDAC inhibition is less beneficial in the context of atherosclerosis.

Together, our data highlight the potential for drugs that selectively target individual HDACs to improve effectiveness in atherosclerosis treatment. In this context, (macrophage-specific) HDAC3 inhibition may be an attractive target for atherosclerosis therapy since its deletion promotes anti-atherogenic macrophage responses while inhibiting inflammatory macrophage cues (Mullican et al., 2011; Kobayashi et al., 2012). Moreover, myeloid HDAC3 deficiency improved collagen deposition and lipid handling in atherosclerotic plaques and induced a more stable plaque phenotype (Hoeksema et al., 2014). Inhibitors that preferentially inhibit HDAC3 also exert antiatherogenic effects *in vitro* (Van den Bossche et al., 2014) but HDAC3-selective drugs (especially macrophage-specific ones) that are applicable *in vivo* are currently not available.

Overall, we demonstrate that targeting HDACs in monocytes and macrophages with ESM technology inhibits both inflammation and monocyte to macrophage differentiation while only minimally affecting atherosclerosis endpoints. While this is an improvement in comparison to the previously applied non-targeted broad-spectrum inhibitor TSA, our study supports the need for drugs that selectively inhibit individual HDACs in target cells.

## Data Availability Statement

The datasets generated for this study are available on request to the corresponding author.

## Ethics Statement

All animal experiments were conducted at the University of Amsterdam and approved (permits: DBC242 and 103169) by the Committee for Animal Welfare of the Academic Medical Center, University of Amsterdam. All animal studies were ethically reviewed and carried out in accordance with European Directive 2010/63/EEC and the GSK Policy on the Care, Welfare and Treatment of Animals.

## Author Contributions

Conceptualization, JB, RL-M, MW, WJ, PM and HL. Methodology, JB, RL-M, RF, SB, SV, MG, CR, AN. Formal Analysis, RL-M, JB, AN, RF, SB, SV, MG and CR. Writing – Original Draft, RL-M, JB. Writing – Review & Editing, RL-M, JB, RF, HL, PM, and MW. Visualization, RL-M, JB. Supervision, MW, PM and JB. Funding Acquisition, MW, WJ, RP and IR. All authors read and approved the final manuscript.

## Funding

Our work is supported by The Netherlands Heart Foundation (CVON 2011/B019, CVON 2017-20 to MW), Spark-Holding BV (2015B002 to MW), the European Union’s Horizon 2020 research and innovation program under Grant Agreement No. ITN-2014-EID-641665 (ITN-grant EPIMAC to MW) and Foundation Leducq (LEAN Transatlantic Network Grant to MW).

## Conflict of Interest

RF, SB, HL, RP, PM, and IR are employees and shareholders at GSK.

The remaining authors declare that the research was conducted in the absence of any commercial or financial relationships that could be construed as a potential conflict of interest.
