# Supplementary material for: Targeting Histone Deacetylases in Myeloid Cells Inhibits Their Maturation and Inflammatory Function With Limited Effects on Atherosclerosis
Source: Front Pharmacol. 2019 Oct 29;10:1242. doi: 10.3389/fphar.2019.01242 (PMC6830127; doi:10.3389/fphar.2019.01242)

Supplementary Material

# Supplementary Tables

**Supplementary Table 1**

**Antibodies for intracellular acetylation mice experiments**

| **Antibody** | **Fluorochrome** | **Clone** | **Species** | **Supplier** |
| --- | --- | --- | --- | --- |
| CD11b | APC | M1/70 | Rat | BD |
| Ly6G | FITC | 1A8 | Rat | BD |
| CD3 | PerCP/Cy5.5 | 500A2 | Syrian hamster | Biolegend |
| B220 | Pacific Blue | RA3-6B2 | Rat | Biolegend |
| CD115 | BV510 | ASF98 | Rat | Biolegend |
| PanAck | PE | I5G10 | Mouse | Biolegend |
| Anti-mouse IgG (minX react) | PE | I5G10 | Goat | Biolegend |

**Supplementary Table 2**

**Antibodies for macrophage activation experiments**

| **Antibody** | **Fluorochrome** | **Clone** | **Species** | **Supplier** | **Dilution** |
| --- | --- | --- | --- | --- | --- |
| CD80 | PE | 16-10A1 | Armenian Hamster | BD Pharmingen | 1:100 |
| CD86 | APC | GL1 | Rat | eBioscience | 1:100 |
| PDL2 | PE | TY25 | Rat | BD Pharmingen | 1:100 |
| CD71 | PE | C2(F2) | Rat | BD Pharmingen | 1:100 |
| CD301 | Alexa Fluor 647 | ER-MP23 | Rat | Serotec | 1:100 |
| Isotype | PE | RTK2758 | Rat | BioLegend | 1:100 |
| Isotype | APC | RTK2758 | Rat | BioLegend | 1:100 |

**Supplementary Table 3**

**Antibodies for macrophage maturation experiments**

| **Antibody** | **Fluorochrome** | **Clone** | **Species** | **Supplier** | **Dilution** |
| --- | --- | --- | --- | --- | --- |
| CD11b | FITC | M1/70 | Rat | eBioscience | 1:100 |
| F4/80 | APC-eFluor780 | BM80 | Rat | eBioscience | 1:100 |
| Ly6C | Alexa Fluor 647 | ER-MP20 | Rat | Serotec | 1:100 |
| CD64 | PE | X54-5/7.1 | Mouse | Biolegend | 1:100 |
| PanAck | PE | I5G10 | Mouse | Biolegend |  |
| Anti-mouse IgG (minX react) | PE | I5G10 | Goat | Biolegend |  |

**Supplementary Table 4**

**Antibodies for intracellular acetylation human experiments**

| **Antibody** | **Fluorochrome** | **Clone** | **Species** | **Supplier** |
| --- | --- | --- | --- | --- |
| CD66 | FITC | BALB/c IgG_2a_, κ | Mouse | BD |
| CD14 | APC | M5E2 | Mouse | BD |
| anti-acetylated lysine | PE | IgG2b, κ | Mouse | Biolegend |
| Isotype control | PE | IgG_2B_ Clone # 133303 | Mouse | R&D system |

##
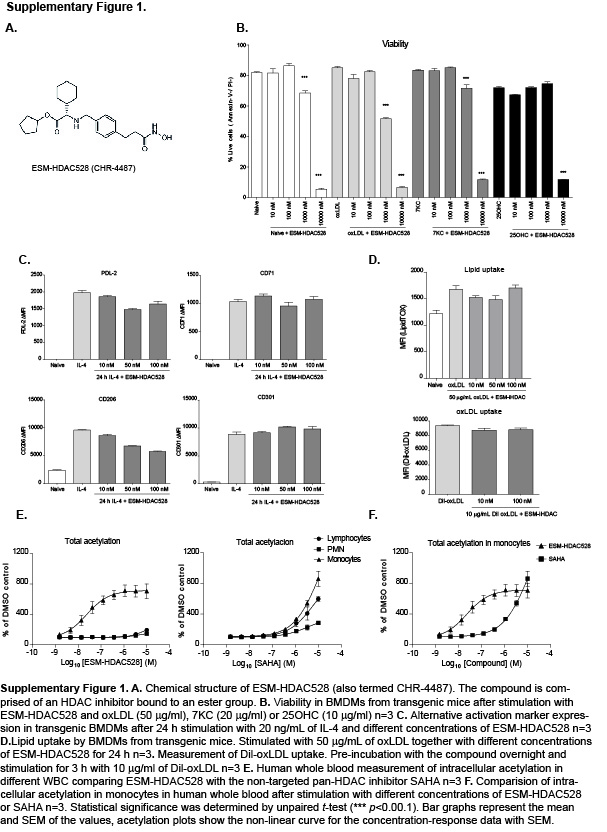

Supplement: Supplementary file 1 [file DataSheet_1.docx]
